# Niraparib Maintenance Therapy for Brain Metastasis in Ovarian Endometrioid Adenocarcinoma With Peritoneal Carcinomatosis: A Comprehensive Case Study and Literature Review

**DOI:** 10.7759/cureus.61355

**Published:** 2024-05-30

**Authors:** Ekaterina Proskuriakova, Barun Aryal, Sarah Khan, Danielle Sanchez, Joseph Moss, Pam Khosla

**Affiliations:** 1 Internal Medicine, Mount Sinai Hospital, Chicago, USA; 2 Hematology and Oncology, Mount Sinai Hospital, Chicago, USA; 3 Internal Medicine, Ross University School of Medicine, Bridgetown, BRB

**Keywords:** endometrioid carcinoma, literature review of disease, case report, metastatic ovarian cancer, niraparib

## Abstract

Brain metastasis is a rare complication of ovarian cancer, always found at the advanced stage. Even though different multimodal approaches are available, including surgical intervention and radiotherapy, there are no official guidelines for handling this serious complication. Poly(adenosine diphosphate-ribose) polymerase (PARP) inhibitors are a group of medications initially used for maintenance therapy in platinum-sensitive recurrent ovarian cancer. Niraparib has shown some efficacy in patients with brain metastasis due to its unique properties of penetrating the blood-brain barrier.

Here, we present the case of a 51-year-old patient with advanced ovarian cancer with no germline breast cancer susceptibility gene (BRCA) mutations. Despite undergoing surgery and multiple rounds of chemotherapy, the patient's condition worsened, culminating in brain metastasis. Given her neurological issues, radiotherapy was not an option, prompting the initiation of a 300 mg dose of niraparib.

To date, only sporadic case reports in the literature have described patients with ovarian cancer treated with niraparib and complicated by brain metastasis. Our case is unique because it is the first case of a patient with the endometrioid type of ovarian cancer.

## Introduction

Ovarian cancer is the fifth most common cause of women's death worldwide and one of the most frequent reasons for death in those diagnosed with gynecological cancers. In 2022, there were around 20,000 newly diagnosed cases and approximately 13,000 fatalities attributed to ovarian cancer in the USA, accounting for 1.2% of all women's cancers. The five-year survival for ovarian cancer has been around 50% in recent years [1].

Ovarian malignancies are presented by several histopathologic variants such as high-grade serous, low-grade serous, endometrioid, clear cell, and mucinous subtypes. Epithelial in origin represents the vast majority of ovarian cancers, accounting for about 90% of all the tumors, with serous carcinoma being the most popular subtype [2]. Ovarian endometrioid carcinoma is the second most common histopathological subtype of epithelial ovarian cancer that accounts for about 8-15% of all ovarian carcinomas and is developed from endometriosis [3]. 

Most of the malignancies are diagnosed in the late stages of the disease (stages III and IV) because ovarian cancer is usually asymptomatic in its early stages. The symptoms usually manifest with the advanced disease with a combination of abdominal bloating, back pain, urinary symptoms, and dyspareunia [4]. Ovarian cancer mainly metastasizes to the peritoneal cavity and via the lymph vessels to the lymphatic nodes. Ovarian cancer rarely metastasizes to the brain, with an estimated incidence of around 5% [5].

Management of ovarian cancer routinely includes chemotherapy and surgery. Initial treatment of patients with advanced tumors is usually debulking surgery performed as laparotomy with total abdominal hysterectomy, bilateral salpingo-oophorectomy (BSO), omentectomy, resection of other tumor deposits, biopsies of the peritoneum, and lymphadenectomy as appropriate [6]. In addition, patients should be evaluated with genetic tests and evaluated for the germline, somatic breast cancer susceptibility gene (BRCA1/2) mutations. Systemic chemotherapy conventionally consists of an intravenous platinum-based regimen (carboplatin or cisplatin) with a bevacizumab maintenance [7]. However, for certain patient populations, such as patients with advanced age (≥70 years) and/or comorbidities, it was shown better clinical outcomes in patients taking carboplatin combination therapy with paclitaxel [8].

Up to 80% of patients with advanced ovarian cancer will have tumor recurrence [9]. After cancer recurrence, platinum-based therapy does not have the same level of sensitivity in these patients. Hence, there is a need for target-directed therapies that can overcome this challenge. One of these medications represents poly(adenosine diphosphate-ribose) polymerase (PARP) inhibitors such as niraparib. These medications initially became important as a maintenance therapy for patients with ovarian cancer. It was shown that they extend progression-free survival (PFS) up to five months in platinum-sensitive recurrent ovarian malignancy compared to placebo [10]. 

We present a case of niraparib use in a 51-year-old patient diagnosed with stage IV endometrioid ovarian cancer with a wild-type BRCA1 mutation. She had a recurrence of her malignancy with cerebral metastasis. The patient was started on niraparib subsequently after her second line of chemotherapy. To our knowledge, there is currently no case report on the successful use of niraparib in patients with cerebral metastasis from endometrioid ovarian cancer. 

## Case presentation

A 51-year-old perimenopausal patient presented to the hospital for abdominal distension and pain in September 2019. She endorsed having night sweats and weight loss with pain near the epigastric umbilicus and supra-pelvic regions radiating to the back. Her abdominal exam was significant for the abdominal distension. Her vitals were stable: her temperature was 98.8°F, her pulse was 104 beats per minute, and her blood pressure was 130/90 mmHg. She was saturating 98% on room air. 

**Figure 1:**
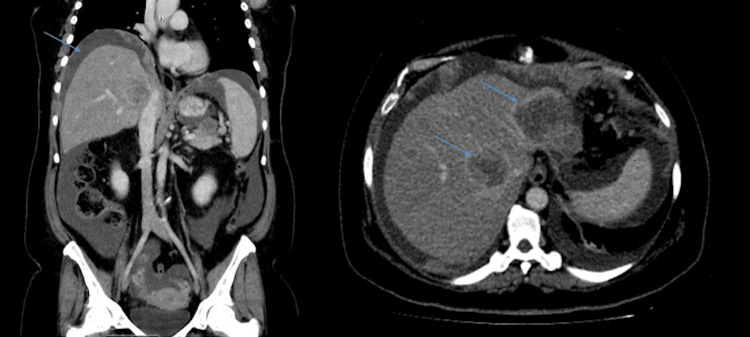
CT of the abdomen and pelvis with contrast series showing vastly diffuse metastatic disease with peritoneal carcinomatosis. The blue arrow on the right shows a moderate to large volume of ascites. Two blue arrows on the right show multiple enhancing, hepatic masses. The left hepatic lobe mass measures 4.9 cm × 4.6 cm. The right hepatic mass measures 3.8 cm × 3.7 cm. CT: computed tomography

The patient had previously been in good health, with no history of hypertension or diabetes and no familial hereditary diseases. Her laboratory results were within normal limits. A computed tomography (CT) scan showed adnexal masses with widespread metastatic disease and extensive peritoneal carcinomatosis (Figure 1). 

Paracentesis was done, with cytology results being highly suspicious for malignancy (Table 1). The genetic test was negative for the pathogenic germline mutation BRCA1. Her cancer antigen (CEA) 125 was elevated at 517 kU/L, carbohydrate antigen (CA) 19-9 was 645 kU/L, and human epididymis (HE) 4 was 37 kU/L. Left axillary lymph node biopsy confirmed metastatic well-differentiated adenocarcinoma. 

**Table 1:** Paracentesis cytology results.

Peritoneal fluid	Results
Amount (mL)	500
Color	Hazy amber fluid
Cytology	Predominant macrophages with single clusters of atypical cells, highly suspicious for malignancy
Immunostains	ER-, MOC-31+, CD68+

**Figure 2:**
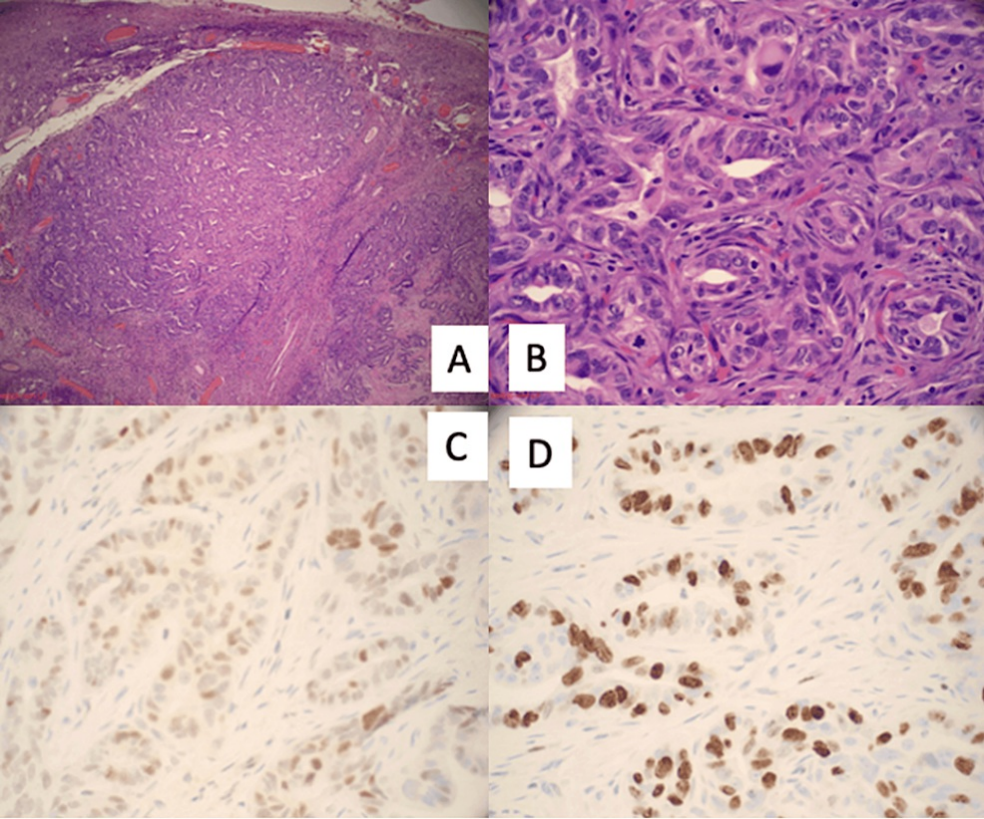
Histology of the surgical specimen. (A) Endometrial carcinoma of the ovary (low-power field, ×10). (B) Endometrial carcinoma of the ovary (high-power field, ×40). (C) Immunostain of p53 (positive). (D) Immunostain of Ki67 (proliferation rate) 80%.

The patient initially underwent two rounds of carboplatin and paclitaxel chemotherapy. A CT scan of the abdomen demonstrated some positive responses to the treatment. The patient wasn't considered a suitable candidate for surgery at that point. In February 2020, five months after she was started on treatment, the patient underwent debulking surgery, and the pathology results revealed the presence of residual ovarian endometrioid adenocarcinoma affecting both ovaries, as depicted in Figure 2. The patient's cancer was staged as IVB according to the International Federation of Gynecology and Obstetrics (FIGO) staging system.

**Figure 3:**
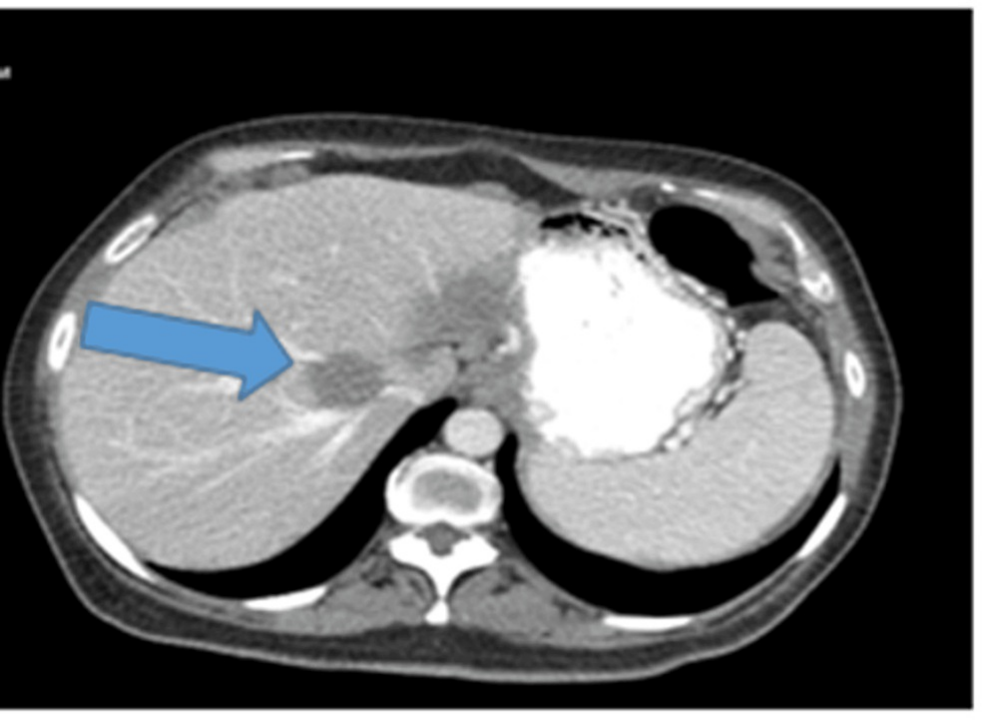
Chest CT abdominal with contrast showed new liver metastasis. CT: computed tomography

The patient underwent carboplatin and gemcitabine for a total of six cycles and was commenced on bevacizumab for maintenance therapy with the subsequent progression of the disease on a CT scan (Figure 3). 

Her genetic testing did not show any actionable mutations, the tumor mutation burden being four mutations per megabase, and SDC4-neuregulin 1 (NRG1) fusion was positive. Therefore, afatinib was added to the patient's bevacizumab maintenance therapy in February 2021. Subsequently, the patient was admitted twice because of hematemesis; bevacizumab was stopped due to the patient developing upper gastrointestinal bleeding because of chronic gastritis and varices secondary to portal hypertension in June 2021. 

For the next 13 months, the patient began to have signs of disease regression with an increase in appetite, weight gain, and mobility. Tumor marker CA-125 was 7 kU/L, and imaging showed a favorable response to therapy. 

However, in July 2022, the patient was found to have progression of the disease with new bone lytic lesions on her CT scan and worsening tumor markers (which was CA-125 kU/L); hence, she commenced chemotherapy with carboplatin and gemcitabine. 

However, her disease continued to progress with further development of bone metastasis on CT, for which she received palliative radiation therapy. The patient's regimen was switched to doxorubicin, and she was started on zoledronic acid for her bone metastasis. 

**Figure 4:**
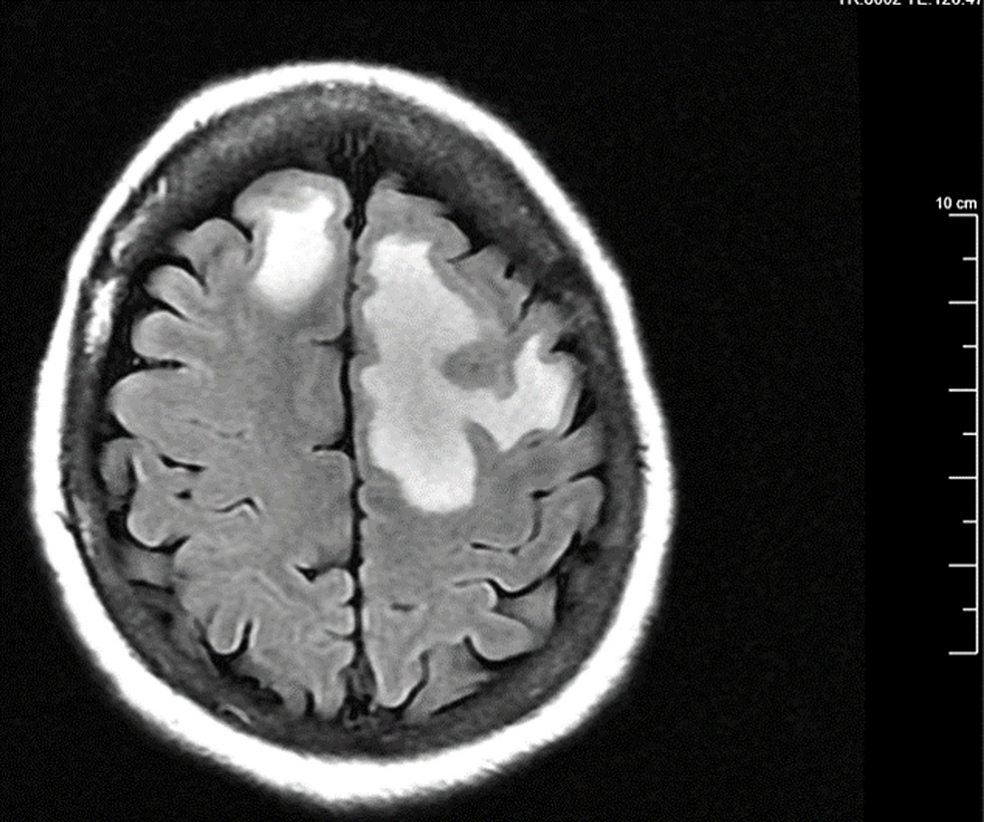
MRI of the brain metastases demonstrates two areas of intra-axial enhancing lesions in the left precentral gyrus and the right frontal lobe with restricted diffusion. MRI: magnetic resonance imaging

On the last day of her third cycle of doxorubicin, she was noted to be confused. On physical examination, she was alert to herself but not to place and time. There were no other positive neurological signs, and the laboratory test findings of tumor markers were all within the normal limits. Her magnetic resonance imaging (MRI) showed two brain lesions with localized mass effect that was not shown before in the previous images (Figure 4). The patient received a dexamethasone 10 mg loading dose with a subsequent switch to 4 mg every six hours. 

**Figure 5:**
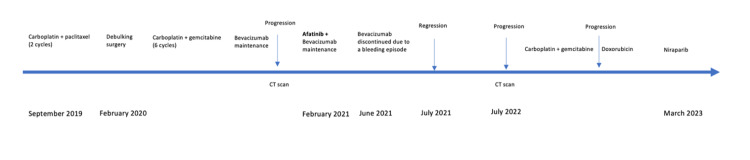
Timeline of different treatments and disease status.

On initial next-generation sequencing (NGS) patients, homologous recombination deficiency (HRD) was noted to be less than 16%. Nevertheless, it was decided to give a trial of the niraparib, given the patient's functional status. Niraparib 300 mg daily was started in March 2023, and weekly full blood count testing was monitored. The patient's weight was 76 lbs during the initiation of the therapy, which is why the dose was reduced to 200 mg daily. The patient was not a good candidate for the stereotactic radiotherapy as she was not able to lay still, having choreiform movements of her upper and lower extremities. The timeline of the patient's progression on different lines of therapy is shown in Figure 5.

## Discussion

Brain metastases are prevalent in the lung, breast cancer, and melanoma, while in patients with ovarian cancer, they are rare and are considered unfavorable regarding patient prognosis. Brain metastasis from the ovaries is mostly found in a widely disseminated and highly progressed disease. The main mechanism of this metastasis is a hematogenous spread of the tumor to the pulmonary system and, later, through the blood vessels of the lungs to the brain [11]. Ogawa et al., in their research, showed that only 0.7% (18 women out of 2,729) developed brain metastasis out of all patients with primary genital cancer who received treatment from 1985 to 2006 [12]. 

The mean team from the initial patient presentation to the development of brain metastasis in patients with ovarian cancer could vary from 11 to 46 months, with the median being about 22 months [13]. LeRoux et al., in their work, demonstrated that the timeframe when patients were diagnosed with brain metastasis was five times shorter in those who presented with ovarian malignancy stage III/IV compared to those who had stage I/II ovarian cancer [14]. In another paper, Cohen et al. showed that those patients with grade 3, poorly differentiated ovarian tumors had a time interval of about 1.5 years between the cancer diagnosis and metastasis. In comparison to grades 1 and 2, moderately differentiated ovarian tumors, the median interval was 4.73 years (P=0.03) [15]. In our case, the patient developed metastasis within 42 months of initial diagnosis.

The most common symptoms of brain metastasis include headache in 50% of patients, confusion, altered mental status, generalized weakness, gait disturbances, neurological deficits, papilledema, and seizures [16]. In our case, the patient developed confusion as a presentation of the brain metastasis.

Currently, no guidelines exist for managing patients with brain metastasis in ovarian cancer. Several suggested approaches are radiotherapy, including whole-brain radiation therapy (WBRT) and gamma knife radiosurgery (GKRS), surgical excision in selected groups of patients, chemotherapy, or their combination [11]. 

WBRT is the first option for brain metastasis from ovarian cancer if there are multiple lesions. Cohen et al. showed that WBRT combined with surgery demonstrated a favorable survival rate of a median of 23 months than WBRT with a median of 5.33 months or surgical management alone with a median of 6.9 months (P<0.01). However, WBRT has a significant number of side effects, including fatigue and memory loss [15]. GKRS is another option for brain metastasis management, especially in patients with a single metastasis and multiple comorbidities and those who are not candidates for surgical interventions. Surgically removing brain metastases can serve as a viable option in place of radiotherapy and might prove beneficial for specific patients in managing intracranial pressure, alleviating symptoms, and enhancing overall survival [17].

PARP inhibitors can treat brain metastasis in ovarian cancer by crossing the blood-brain barrier [18]. Table 2 demonstrates four case reports have reported the successful use of PARP inhibitors in the setting of brain metastasis in patients with ovarian cancer [19-22]. However, there was no case report where the patient was treated for brain metastasis secondary to the endometrioid type of ovarian cancer. 

**Table 2:** Four cases of successful treatment for brain metastases with PARP inhibitors. PARP: poly(adenosine diphosphate-ribose) polymerase

Authors	Type of tumor	Gene mutations	PARP inhibitor
Bangham et al. 2016 [19]	High-grade serous ovarian cancer	BRCA2, CK7, WT1, CA-125	Olaparib
Sakamoto et al. 2019 [20]	Primary peritoneal cancer	BRCA1	Olaparib
Gallego et al. 2021 [21]	High-grade serous ovarian carcinoma	BRCA1	Olaparib
Gray et al. 2019 [22]	High-grade serous ovarian carcinoma	BRCA1	Niraparib

PARP inhibitors have changed the therapeutic approach in patients with BRCA-positive ovarian cancer. PARP is a non-histone protein that recognizes and repairs single-stranded DNA breaks. If the cell obtains a single-stranded DNA break, it will subsequently lead to the double-stranded break that could be repaired via the homologous recombination (HR) mechanism by exchanging nucleotide sequencing between DNA molecules. Dysfunction of both repair mechanisms, single-stranded via PARP and double-stranded via HR in the cell, is called "synthetic lethality" [23]. BRCA is a tumor suppressor gene that expresses a protein that repairs double-stranded DNA breaks via HR. Patients' cells with the mutation in the BRCA gene will not be able to repair their cells via the HR mechanism, and therefore, they are considered to have HRD [24]. PARP inhibitors can block single-stranded DNA repair, leading cells to synthetic lethality and, subsequently, apoptosis.

In 2014, olaparib was the first PARP1/2 inhibitor approved by the United States Food and Drug Administration (US FDA) for treating breast cancer and ovarian cancer with BRCA1/2 defects [25]. Later, in 2016, another PARP1/2, rucaparib, was approved for patients with BRCA1/2 mutations for the treatment of ovarian cancer with the previous more than two lines of chemotherapies [26]. One year later, in 2017, niraparib was approved for patients with and without BRCA1/2 mutations for recurrent ovarian cancer and maintenance therapy [10]. In 2018, talazoparib, a PARP1 inhibitor, was approved for patients with BRCA mutations for the treatment of locally advanced or metastatic HER-2-negative breast cancer [27].

In the NOVA trial, niraparib was evaluated in 576 patients with ovarian cancer with and without BRCA for the maintenance therapy for platinum-sensitive recurrent ovarian cancer in the dose of 300 mg daily. In patients taking niraparib, irrespective of the BRCA status, PFS was significantly longer compared to the placebo. The median PFS in the BRCA-positive cohort taking niraparib was 21 months compared to 5.5 months in the placebo group with a hazard ratio (HR) of 0.27 and a 95% confidence interval (CI) of 0.17-0.41 (P<0.001). In the BRCA-negative group in HRD tumors, the median PFS was 9.3 months and 3.9 months in niraparib and placebo groups, respectively (HR: 0.38; 95% CI: 0.24-0.59; P<0.001). In patients without HRD and BRCA-negative tumors, the median PFS in the niraparib group was 9.3 months, and it was 3.9 months in the placebo group (HR: 0.45; 95% CI: 0.34-0.61; P<0.001). The most common adverse effects were thrombocytopenia and anemia [10].

In a preclinical investigation using tumor models, it was determined that niraparib could penetrate the blood-brain barrier, leading to an increased drug concentration in brain tissues and enhanced tumor-suppressing capabilities. Conversely, this outcome was not witnessed in the group receiving olaparib [18]. Given these discoveries and the examination results indicating a BRCA1 mutation, we opted for niraparib as the treatment approach for our patient.

Additional research is needed to understand better the mechanisms behind the development of brain metastases in patients with recurrent ovarian cancer and to determine the most appropriate treatment approaches. The effectiveness of PARP inhibitors, whether used alone, sequentially, or in combination with brain radiotherapy, remains uncertain. Given the rarity of this occurrence, the prospects of conducting clinical trials are limited, underscoring the importance of reporting clinical cases and establishing international registries to enhance our understanding in this area. Furthermore, it is essential not to exclude patients with ovarian cancer and controlled brain metastasis from clinical trials to gain insights into how new drugs perform in these individuals.

## Conclusions

While brain metastasis from ovarian cancer is an infrequent event, it carries a grim prognosis and substantial deterioration in a patient's quality of life. The choice of niraparib for the patient in this case report was based on prior in vitro research that confirmed niraparib's capability to penetrate the blood-brain barrier. The patient initially experienced a transient improvement in her condition after commencing niraparib treatment; however, she later traveled to Mexico and was lost to further medical monitoring.
